# Granulome conjonctival suite à un traumatisme par épine végétale négligé: à propos d’un cas

**DOI:** 10.11604/pamj.2016.25.8.10477

**Published:** 2016-09-16

**Authors:** Taha Elghazi, Amine Eljai, Maryama Elkaddoumi, Omar Lazrek, Sofia Hassani saoudi, Taib Belkbir, Abdellah Amazouzi, Lalla Ouafae Cherkaoui, Rajae Daoudi

**Affiliations:** 1 Université Mohammed V Souissi, Service d’Ophtalmologie A de l’Hôpital des Spécialités, Centre Hospitalier Universitaire, Rabat, Maroc

**Keywords:** Granulome conjonctival, épine végétale, traumatisme pénétrant, Conjunctival granuloma, thorn, penetrating trauma

## Abstract

Il est parfois difficile de détecter un corps étranger végétal intraoculaire, principalement dans les cas où l'histoire du traumatisme oculaire n'est pas claire, ou chez les patients qui consultent plusieurs mois après le traumatisme. Nous rapportons un cas rare d'un enfant de 7 ans qui s'est auto traumatisé au niveau de l'œil gauche par une épine végétale de cactus, 3 mois avant son admission, ce qui a entraîné un granulome conjonctival temporal supérieur avec réaction inflammatoire du segment antérieur. L'exploration chirurgicale a été réalisée permettant l'extraction de l'épine avec une exérèse totale du granulome. Une légère amélioration clinique et de l'acuité visuelle a été notée puis le globe oculaire a évolué vers la phtyse quelque mois après l'extraction.

## Abstract (en)

It is sometimes difficult to detect a plant intraocular foreign body, mostly in cases where the history of ocular trauma is unclear, or in patients who consult several months after the trauma. We report a rare case of a 7 year old child with self inflicted trauma of the the left eye by a cactus thorn 3 months before admission, which resulted in a temporal conjunctival granuloma with higher inflammatory reaction of the anterior segment. Surgical exploration was performed allowing the extraction of the thorn with total excision of the granuloma. A slight clinical improvement in visual acuity was observed, but the eyeball secondarily atrophied few months after extraction.

## Introduction

Les granulomes conjonctivaux sont des entités rares qui peuvent survenir dans des contextes diverses, post-chirurgicale, infectieuses ou à corps étrangers (CE). Ces derniers peuvent avoir une origine post-traumatique ou spontanément sans que le patient ait la notion d'exposition ou de pénétration de corps étranger [1]. Le pronostic fonctionnel de l'œil peut être mis en jeu par ces corps étrangers végétaux intraoculaires et entraîner des complications potentiellement graves [2]. Nous rapportons ici un cas inhabituel d'un enfant de sept ans qui a développé un granulome conjonctival à corps étranger au niveau de l'œil gauche suite à un traumatisme par épine de cactus qui est resté négligé pendant 3mois.

## Patient et observation

**Figure 1:**
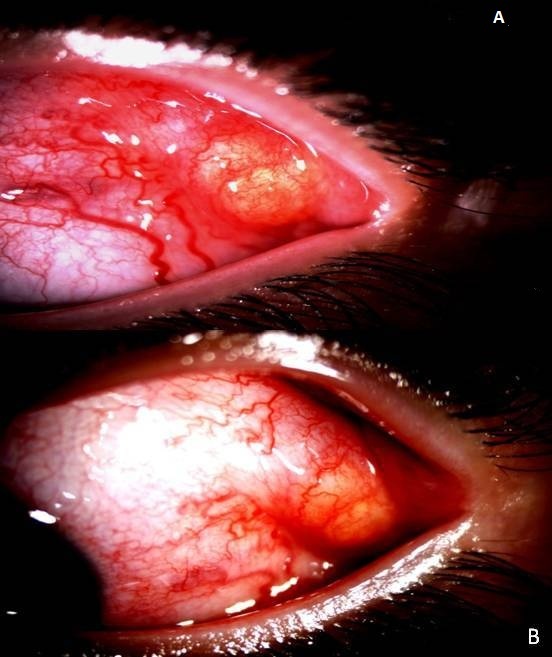
A) granulome conjonctival; B) sa localisation par rapport au limbe

**Figure 2:**
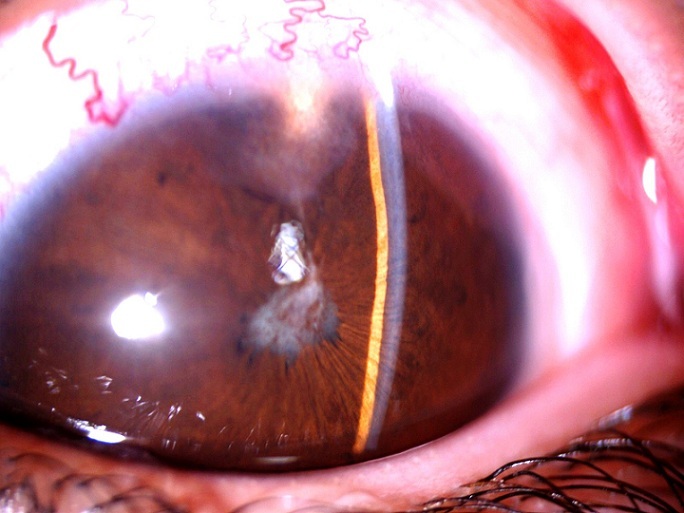
Segment antérieur montrant la chambre réduite, la sécclusion pupillaire et la membrane de fibrine tapissant l’iris en supérieur

**Figure 3:**
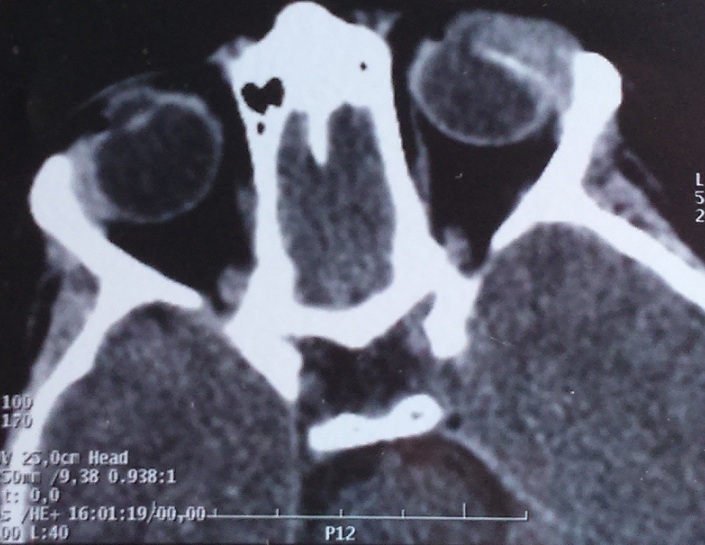
Coupe scannographique axiale montrant l’épine végétale associée au granulome conjonctival

**Figure 4:**
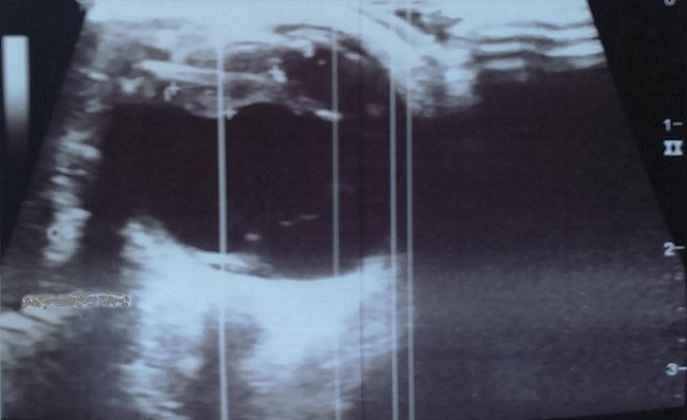
Échographie oculaire montrant l’épine végétale entourée de granulome conjonctival

**Figure 5:**
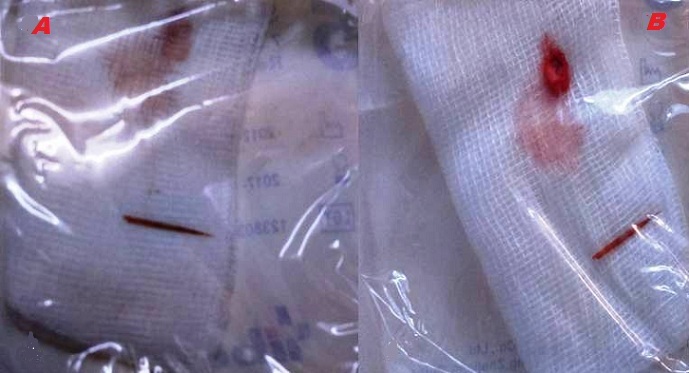
A) épine végétale après extraction chirurgicale; B) le granulome conjonctival adressé pour examen anatomopathologique

Il s'agit d'un enfant de 7ans qui a comme antécédents une notion de traumatisme oculaire par épine végétale de cactus 3 mois avant son admission. Il a été amené aux urgences ophtalmologiques suite à la découverte fortuite par les parents d'une excroissance au niveau de la conjonctive bulbaire en temporal supérieur de l'œil gauche. L'examen ophtalmologique trouve une acuité visuelle à mouvement des doigts au niveau de l'œil gauche et à 10/10 à l'œil droit. L'examen des annexes note un léger œdème palpébral avec hyperhémie conjonctivale surtout temporale et la découverte lors du regard en adduction d'une tuméfaction conjonctivale blanchâtre irrégulière en temporal supérieur de 5mm*5mm*2mm de taille entourée d'une réaction inflammatoire (Figure 1). La cornée est claire. L'examen du segment antérieur montre une chambre antérieure réduite de profondeur, une sécclusion pupillaire, un iris bombé et une membrane de fibrine tapissant l'iris en supérieur. Le cristallin note une cataracte totale blanche (Figure 2). Le fond d'œil est inaccessible. L'examen de l'œil droit est normal. Un bilan radiologique fait de TDM orbitaire (Figure 3) et d'échographie oculaire (Figure 4) a été demandé en urgence. L'exploration chirurgicale a permis l'extraction du corps étranger (épine végétale de cactus mesurant 10mm) avec exérèse du granulome conjonctival adressé pour examen anatomo-pathologique (Figure 5). L'évolution a été marquée par une légère amélioration de l'acuité visuelle passant de voit bouger les mains à compte les doigts à 50cm avec diminution de la réaction inflammatoire de la chambre antérieure et le bombement de l'iris puis le globe oculaire a évolué vers la phtyse vu l'ancienneté du traumatisme. L'examen anatomopathologique a mis en évidence un foyer granulomateux nodulaire recouvert par l'épithélium conjonctival. Ce granulome est composé de cellules macrophagiques, de cellules géantes et d'autres cellules de l'inflammation chronique comme les lymphocytes.

## Discussion

Les traumatismes oculaires représentent un véritable problème de santé publique. Selon une étude réalisée au Maroc en 1992 et intéressant toute la population (adultes et enfants), les traumatismes sont responsables de 1.3% des cécités bilatérales et 14% des pertes de vision unilatérale [3]. Les agressions sont responsables de 18 % des corps étrangers (CE) intraoculaires. Il faut souligner que 16 % des plaies oculaires de l'enfant sont induites par un CE, le plus souvent lors d'activité de jeux ou par projections diverses [4]. Par ailleurs, devant tout traumatisme oculaire, un corps étranger (CE) doit être suspecté, quelle que soit sa localisation dans l'œil. Différents caractéristiques doivent être déterminées pour comprendre sa position, les risques encourus et définir la conduite à tenir thérapeutique : sa nature, son origine avec sa taille, sa forme, son énergie, sa température, sa trajectoire probable et le risque de contamination microbienne [5]. La tomodensitométrie et l'échographie oculaire sont les examens à réaliser en première intention mais ils peuvent être faussement négatifs : en particulier, l'hypodensité du corps étranger en bois à la tomodensitométrie peut être confondu avec de l'air [6]. L'IRM peut être utile dans un contexte de traumatisme quand un corps étranger végétal est suspecté notamment si les autres examens sont négatifs [7]. Les granulomes conjonctivaux peuvent survenir dans différents contextes pathologiques (post-traumatique et infectieux) ou spontanément. Les granulomes conjonctivaux à corps étranger ont été décrits pour la première fois en 1861 par Schön sous le terme de « conjonctivite nodulaire », en réaction à l'exposition aux poils de chenille [1]. De nombreux corps étrangers ont été rapportés depuis : cils, cheveux, fibres textiles naturelles ou synthétiques [8].

En effet, dans une étude réalisée sur des prélèvements post-mortem chez des adultes, Saer et al. ont évalué la prévalence des granulomes conjonctivaux à corps étranger à 10 % environ [9]. Ce type de réaction à corps étranger peut être soit symptomatique, s'il existe une réaction inflammatoire locale marquée depuis plusieurs semaines à plusieurs mois [1, 10]. Certains auteurs expliquent la faible réaction inflammatoire dans certains cas par l'absence de surface très irritante au niveau du corps étranger et par l'inclusion de celui-ci dans le mucus, diminuant d'autant plus son pouvoir agressif [11]. Ceci peut expliquer le retard de consultation chez notre patient vu son âge très jeune et la localisation du corps étranger par rapport au limbe. Du fait de leur présentation clinique variable et aspécifique sous la forme d'une tuméfaction conjonctivale plus ou moins inflammatoire, les diagnostics différentiels sont nombreux (granulome pyogénique, conjonctivite nodulaire, conjonctivite ligneuse, sarcoïdose, dermolipome et rhabdomyosarcome) [11]. Il faut insister sur le risque de survenue d'une endophtalmie post traumatique qui peut s'élever à environ 30% des patients dans les suites d'un traumatisme à globe ouvert surtout négligé. Les principaux facteurs de risque sont la présence d'un corps étranger, l'ouverture de la cristalloïde, la pénétration intraoculaire d'un objet souillé et, par conséquent, la survenue de traumatisme en milieu rural comme le cas de notre patient. La fréquence de survenue d'une infection en présence d'un CE intraoculaire (CEIO) varie entre 10 et 25% des cas selon les études [5]. L'endophtalmie post traumatique diffère de l'endophtalmie post opératoire tant sur le plan du spectre microbien que du pronostic fonctionnel. Le pronostic visuel peut être plus sombre par une prise en charge retardée. Il est de plus fonction de la nature du traumatisme et des lésions induites, ainsi que de l'agent infectieux en cause. Un prélèvement endoculaire d'humeur aqueuse et de vitré pour examen microbiologique (culture et polymerase chain reaction [PCR]) doit être réalisé suivies d'injections intravitréene d'antibiotiques à large spectre plus ou moins d'antifongiques [5, 12]. Une antibiothérapie à large spectre doit être instituée systématiquement après les traumatismes pénétrants par objets en bois [13]. La prise en charge chirurgicale doit être confiée à des équipes spécialisées car le risque chirurgical n'est pas négligeable. La voie d'abord chirurgicale dépend de la localisation du corps étranger, guidée par l'imagerie [13, 14].

## Conclusion

Du fait de leur présentation clinique inhabituel comme l'aspect particulier chez notre patient, les granulomes conjonctivaux à corps étrangers restent très probablement sous-estimés. Il est toujours important de déterminer l'existence ou non d'un CE intraoculaire et ceci par un interrogatoire attentif. L'examen minutieux à la lampe à fente et les examens complémentaires sont indispensables pour affirmer sa présence afin de ne pas retarder la prise en charge et de prévenir la survenue de complications qui peuvent compromettre le pronostic visuel.
